# Impact of Exposure to Ambient Fine Particulate Matter Pollution on Adults with Knee Osteoarthritis

**DOI:** 10.3390/ijerph18189644

**Published:** 2021-09-13

**Authors:** Hongbo Chen, Junhui Wu, Mengying Wang, Siyue Wang, Jiating Wang, Huan Yu, Yonghua Hu, Shaomei Shang

**Affiliations:** 1 Department of Epidemiology and Biostatistics, School of Public Health, Peking University, No. 38 Xueyuan Road, Beijing 100191, China; chenhongbo@bjmu.edu.cn (H.C.); jhsophie@163.com (J.W.); mywang@bjmu.edu.cn (M.W.); siyue.wang@pku.edu.cn (S.W.); jiating@pku.edu.cn (J.W.); yuhh@pku.edu.cn (H.Y.); 2 School of Nursing, Peking University, No. 38 Xueyuan Road, Beijing 100191, China; 3 Medical Informatics Center, Peking University, No. 38 Xueyuan Road, Beijing 100191, China

**Keywords:** knee osteoarthritis, fine particulate matter, air pollution, outpatients, time-series, China

## Abstract

The impact of exposure to fine particulate matter (PM_2.5_) on the incidence of knee osteoarthritis is unclear, especially in Beijing which is a highly polluted city. We conducted a time-series study to examine the correlation between PM_2.5_ exposure and outpatient visits for knee osteoarthritis in Beijing. Changes (in percentage) in the number of outpatient visits corresponding to every 10-μg/m^3^ increase in the PM_2.5_ concentration were determined using a generalized additive quasi-Poisson model. There were records of 9,797,446 outpatient visits for knee osteoarthritis in the study period from 1 January 2010 to 31 December 2017. The daily concentration of PM_2.5_ was 86.8 (74.3) μg/m^3^ over this period. A 10-μg/m^3^ increase in PM_2.5_ concentrations on lag days 0–3 was associated with a 1.41% (95% confidence interval: 1.40–1.41%) increase in outpatient visits for knee osteoarthritis. Females and patients aged above 65 years were more sensitive to the adverse effects of PM_2.5_ exposure. The present findings demonstrate that short-term exposure to PM_2.5_ resulted in an increase in the number of outpatient visits for knee osteoarthritis in Beijing. The findings shed light on the effects of air pollution on knee osteoarthritis and could guide risk-mitigating strategies in cities such as Beijing.

## 1. Introduction

Knee osteoarthritis is a common joint disease that is associated with pain, loss of function and disability, and reduced quality of life [1]. Knee osteoarthritis affects approximately 250 million people globally, and the global prevalence of symptomatic knee osteoarthritis is 10.0–16.0% in adults aged above 60 years [2,3,4,5]. In 2016, China ranked tenth with regard to the number of years lived with disability (YLDs) as a result of osteoarthritis [6], and the Nordic nations ranked fifth with regard to the rate of increase in YLDs associated with osteoarthritis [7]. In fact, knee osteoarthritis is the most common kind of osteoarthritis, accounting for 87% of YLDs associated with osteoarthritis. In China, the number of total knee arthroplasty cases in 2018 was 249,000, which were associated with a total cost of about 12.948 billion yuan [8], making this disease a serious burden for the healthcare system. Therefore, it is important to understand the causative factors and mitigate the risk of knee osteoarthritis in the Chinese population.

The 2015 Global Burden of Diseases, Injuries, and Risk Factors Study (GBD 2015) identified air pollution as one of the main causes of the increase in global disease burden, especially in low- and middle-income countries [9]. Accordingly, in the last few years, mounting evidence has linked indoor/outdoor air pollution to musculoskeletal disease [10,11,12,13], and previous epidemiological studies have shown that environmental factors, especially environmental pollution, may be specifically associated with an increased risk of knee osteoarthritis [14,15]. With regard to the underlying mechanism, air pollutants may cause oxidative stress in the lung, and this may lead to the production of a large number of antibodies and pro-inflammatory cytokines. Alternatively, air pollutants could also be genotoxic and induce epigenetic changes that may lead to cartilage erosion and synovial inflammation [16] and eventually cause the development of musculoskeletal disease [14]. In particular, fine particulate matter or PM_2.5_, which is defined as particulate matter that has an aerodynamic diameter of ≤2.5 μm, is the most harmful to human health, and it ranks fifth in the list of mortality risk factors [17]. Studies have confirmed that PM_2.5_ is a risk factor for heart failure, asthma, ischemic stroke, allergic rhinitis, and other diseases [18,19,20,21]. In terms of musculoskeletal diseases, previous studies have indicated that exposure to high levels of PM_2.5_ could increase the risk of osteoarthritis [15,22]. However, most of these previous studies used cell lines or animal models, or small population samples. Thus, there is a lack of large-scale population studies that explore the effect of PM_2.5_ exposure on the risk of osteoarthritis.

As one of the largest developing countries in the world, China is witnessing accelerated industrialization and urbanization, which have led to a surge in air pollution. In 2016, the majority, that is, 81% of China’s population was exposed to PM_2.5_ concentrations higher than the limit of 35% μg/m^3^ set by the State Environmental Protection Administration [23]. In 2017, 8,51,660 (7,12,002–9,90,271) deaths in China were attributable to PM_2.5_ pollution [24]. The air pollution crisis is particularly severe in Beijing [25] and needs to be addressed urgently. Therefore, this time-series study investigates whether short-term exposure to outdoor PM_2.5_ is correlated with the number of outpatient visits for knee osteoarthritis in Beijing, China. We believe that this is the first such large-scale study on the association between PM_2.5_ exposure and knee osteoarthritis in China.

## 2. Materials and Methods

### 2.1. Data on Outpatient Visits for Knee Osteoarthritis

We collected outpatient data on visits for knee osteoarthritis between 1 January 2010, and 31 December 2017, from Beijing’s Medical Claims for Employees (BMCDE) database. The database contains records of the medical claims of patients who have been availed of Urban Employee Basic Medical Insurance. Medical insurance for urban employees is the main type of medical insurance in Beijing, since rural inhabitants only form a small percentage of the population and the rate of employment is high. By the end of 2017, the number of beneficiaries included in the BMCDE database was 17.7 million, which is equivalent to nearly 80% of the city’s permanent residents. The database includes demographic data (sex and age), medical records (hospital name, hospital level, and dates of consultation), diagnosis in Chinese and diagnosis according to the criteria of the International Classification of Diseases (10th Revision [ICD-10]), diagnosis dates, and treatment costs. Detailed information from the database can be found in previous relevant studies [26,27].

This study is exempt from ethical approval since the BMCDE database does not contain any identifying information about the beneficiaries, and any traceable information we used was encrypted.

### 2.2. Environmental Data

Temperature and humidity data were obtained from the Chinese Meteorological Bureau. Hourly data were obtained for PM_2.5_ concentration from an open-source report released by the US Embassy. According to previous studies, the PM_2.5_ levels monitored by the US Embassy are roughly representative of the levels in the whole city, and can, therefore, be considered as proxy values for population exposure within a range of 40 km of the monitoring station [19,20,28]. This radius covers the majority of the tertiary hospitals, that is, 97.8% (44/45), and also the majority of the secondary hospitals (79.3% [69/87]) [29]. Previous literature has demonstrated the reliability of data from monitoring sites of the US Embassy [19,30]. Real-time data on PM_2.5_ concentrations were published by the national air quality monitoring network of China only from 2013. As a result, for the purpose of this study, the data were only obtained from the US Embassy. Specifically, we examined the correlation of the daily average concentration of PM_2.5_ with the number of daily visits for knee osteoarthritis according to meteorological conditions.

### 2.3. Statistical Analysis

We conducted a time-series analysis of PM_2.5_ exposure and outpatient visits for knee osteoarthritis (adjusted for meteorological factors). According to previous methods used for exploring the relationship between air pollution and health, a generalized additive quasi-Poisson model with the following formula was applied [29,31,32].

$$\mathrm{Log}\left[E\left(Y_{t}\right)\right]=\alpha +{\beta \mathrm{PM}}_{2.5}+\mathrm{public}\;\mathrm{holiday}+\mathrm{day}\;\mathrm{of}\;\mathrm{the}\;\mathrm{week}+\mathrm{ps}\left(\mathrm{calendar}\;\mathrm{time},7\;\mathrm{per}\;\mathrm{year}\right)+\mathrm{ps}\left(\mathrm{temperature},6\right)+\mathrm{ps}\left(\mathrm{relative}\;\mathrm{humidity},3\right)$$

In the above equation,$\;E\left(Y_{t}\right)$ is the anticipated number of daily adult outpatient visits for knee osteoarthritis on day t, and ps () represents a penalized spline function. Public holidays and the day of the week were considered as categorical variables and were adjusted for. β denotes the log value of the relative risk of knee osteoarthritis-related morbidity for every unit rise in the concentration of PM_2.5_, and α denotes the intercept term. In order to verify the robustness of the results, we set various degrees of freedom for calendar time, relative humidity, and temperature based on findings from previous studies, and conducted sensitivity analysis on the results for different degrees of freedom [31].

Separate models were created for each lag day, from the day of outpatient visit (lag 0 days) to three days before the visit (lag 3 days), as well as multiple-day lags (lag 0–1 day, lag 0–2 days, and lag 0–3 days) to explore the exposure–response association between PM_2.5_ and outpatient consultations for knee osteoarthritis [33]. Stratified analyses were conducted to detect the impact of age, sex, and meteorological conditions on knee osteoarthritis risk [34].

The “mgcv” and “nlme” packages of R3.2.2 (R Foundation for Statistical Computing, Vienna, Austria) were used for the statistical analyses in our study. Percentage change in the daily number of outpatient visits for knee osteoarthritis corresponding to a 10 μg/m^3^ increase in the PM_2.5_ concentration was expressed as a percentage with the 95% confidence interval (CI). A two-tailed *p* value of <0.05 was considered to indicate statistical significance. Categorical variables were represented by their percentage values, and continuous variables, by their mean ± standard deviation values.

## 3. Results

### 3.1. Demographic Information

Table 1 presents the demographic data for outpatient visits for knee osteoarthritis. A total of 9,797,446 adult knee osteoarthritis outpatient visits were observed in the BMCDE database between 1 January 2010, and 31 Demcember 2017, and 59.87% of the consultations were recorded in the cool season, that is, November to April. Additionally, 63.61% of the outpatients were female and 63.84% were elderly (≥65 years)

### 3.2. Daily Values of PM_2.5_, Outpatient Visits for Knee Osteoarthritis, Temperature, and Humidity

Table 2 presents the mean (SD) values for daily knee osteoarthritis outpatient visits, PM_2.5_ concentration, temperature, and relative humidity. The daily number of outpatient visits was 3354 (3700), and it ranged from 108 to 30,125. The daily PM_2.5_ value was 86.8 (74.3) μg/m^3^, and it ranged from 1.0 μg/m^3^ to 537.3 μg/m^3^. Over the study period of 2922 days, the daily PM_2.5_ value was below the limit of 25 µg/m^3^ set by the air quality guidelines of WHO only on 509 (17.4%) days [35]. The daily temperature was 14.6 °C (11.3 °C), and relative humidity, 51.8% (20.2%).

### 3.3. Changes in the Number of Outpatient Visits According to PM_2.5_ Concentration

Table 3 shows the percentage changes in outpatient visits for knee osteoarthritis corresponding to each 10-μg/m^3^ rise in PM_2.5_ concentration for different lag days and intervals. The data were adjusted for temperature, relative humidity, day of the week, and public holidays, and the results showed that a 10-μg/m^3^ increase in PM_2.5_ corresponded to an increase of 1.20% (95% CI, 1.20–1.21%) in the number of outpatient visits on the same day. A lag of one day resulted in an increase of 0.61% (95% CI, 0.61–0.62%) in the daily number of visits, and a lag of two days was associated with an increase of 0.59% (95% CI, 0.59–0.59%). Furthermore, based on the percentage increase in outpatient visits for different lag intervals, the PM_2.5_ concentration was found to have a significant cumulative effect that was the highest for a lag interval of zero to three days (1.41%; 95% CI, 1.40–1.41%). These findings indicate a strong exposure-response relationship between the PM_2.5_ concentration and daily number of outpatient visits for knee osteoarthritis.

**Figure 1:**
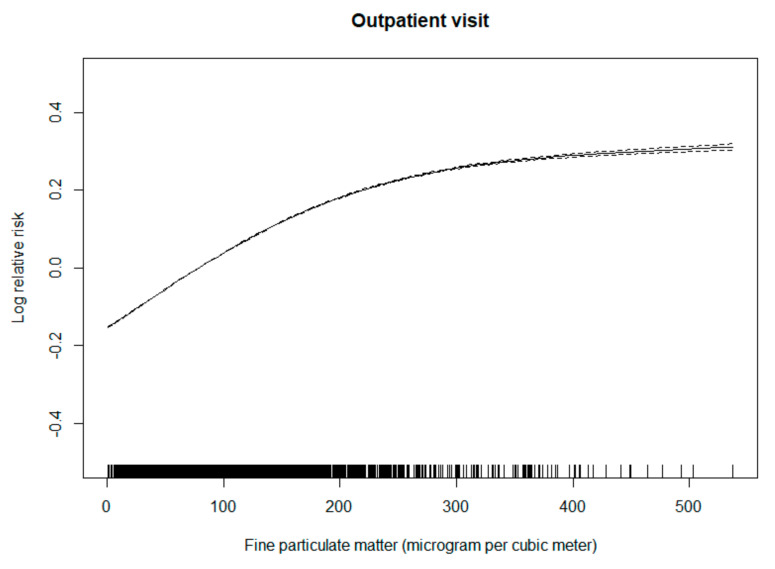
Exposure-response relationship of PM_2.5_ concentrations with the number of daily outpatient visits for knee osteoarthritis. The concentration-response curve (solid line) is shown for the three degrees of freedom of the daily mean concentrations of PM_2.5_ and the log-transformed value of the relative risk (after adjustment for temperature, relative humidity, public holiday, and day of the week) of outpatient visits for knee osteoarthritis between 1 January 2010, and 31 December 2017, in Beijing, China. The dotted lines indicate the 95% confidence intervals.

Figure 1 shows the exposure-response relationship of PM_2.5_ concentrations with the number of daily outpatient visits for knee osteoarthritis. A clear concentration-response curve was observed for PM_2.5_ and outpatient visits for knee osteoarthritis.

### 3.4. Influence of Age, Gender, and Season on Changes in the Number of Outpatient Visits

As shown in Table 4, the association between PM_2.5_ and knee OA outpatient visits was greater in patients aged 65 and over (1.59%, 95% CI: 1.58–1.60%) than in younger age groups (1.28%, 95% CI: 1.28–1.29%). Additionally, the estimated effect of PM_2.5_ was lower in men (1.36%, 95% CI: 1.35–1.37%) than in women (1.41%, 95% CI: 1.40–1.42%). With regard to seasonal influence, the estimated value was higher in the warm season (2.76%, 95% CI: 2.75–2.78%).

### 3.5. Reliability of the Data

Sensitivity analysis (Table 5) showed that after the introduction of variations in the degree of freedom for calendar time, temperature, and relative humidity, the estimated change in the number of outpatient visits did not change significantly. Thus, the relationship derived between PM_2.5_ and outpatient visits for knee osteoarthritis was reliable.

## 4. Discussion

The present study was the first large-scale one conducted in Beijing, China, on the short-term effects of PM_2.5_ exposure on outpatient visits for knee osteoarthritis in China. After some confounding variables, including temperature, relative humidity, day of the week, calendar time, and public holidays, were adjusted for, every 10-μg/m^3^ rise in PM_2.5_ led to a 1.41% (95% CI = 1.40–1.41%) increase in the number of outpatient visits when there was a lag of zero to three days between the exposure and the visit. This effect of exposure to PM_2.5_ was prominent in women and patients over 65 years.

There are very few studies about the impact of air pollution on the incidence of musculoskeletal diseases such as knee osteoarthritis and osteoporosis. According to one such report from Taiwan, individuals who are more likely to be exposed to air pollution have higher chances of developing osteoarthritis [15]. Another study conducted in the United States showed that the likelihood of hospital admissions for bone fractures at osteoporosis-related sites was higher in areas that had greater PM_2.5_ levels [11]. In the context of China, a national-level longitudinal prospective study reported that adults over 45 years who have been exposed to indoor air pollution over a long time have an increased risk of developing arthritis [13]. Further, a cross-sectional survey in China’s Henan Province showed that every 1-μg/m^3^ rise in PM_1_, PM_2.5_, PM_10_, and NO_2_ resulted in a 14.9%, 14.6%, 7.3%, and 16.5% higher risk of osteoporosis [12]. Similar findings have been reported in other studies too [36,37]. Earlier studies have indicated that a large proportion of patients with hip or knee osteoarthritis have osteoporosis too; thus, osteoporosis and osteoarthritis share similar mechanisms and epidemiology [11,38,39]. However, the abovementioned studies are limited by their small sample size and diagnostic methods, which might indicate limited statistical power. In contrast to these studies, our study uses a large sample size that is representative of the working population of Beijing and, therefore, makes an important contribution to the literature on this topic.

The mechanisms via which PM_2.5_ induces osteoarthritis pathways are unclear. One of the main reasons for the increase in outpatient visits after short-term PM_2.5_ exposure could be the increase in knee osteoarthritis-associated pain that results from serious air pollution [40]. With regard to the pathophysiological mechanisms, particulate matter may induce the production of reactive oxygen species [41], which could lead to systemic inflammation and the release of proinflammatory cytokines, such as TNF-α, IL-1β, and IL-6 [42,43]. In particular, IL-1β and TNF-α can stimulate IL-6 expression, and IL-6 is a biomarker reflecting the severity of osteoarthritis [44,45]. Furthermore, an animal experiment on a rat model showed that exposure to particulate matter affects osteocalcin, cartilage oligomeric matrix protein, and N-telopeptides of type I collagen, resulting in decreased bone density, cartilage wear and structural damage, and development of osteoarthritis [14]. Unfortunately, no study has specifically examined the mechanisms by which PM_2.5_ increases the risk of or exacerbates osteoarthritis.

With regard to the variables that influence the number of visits for knee osteoarthritis after PM_2.5_ exposure, we found that being over 65 years old was a strong risk marker. Similarly, a study in Taiwan showed that in terms of the outpatient visit rate for musculoskeletal diseases, people over 65 years old are more likely to be affected by air pollution than those in younger age groups [15]. Based on these findings, it is recommended that elderly people living in Beijing restrict outdoor activities or take protective measures when going out to reduce exposure during periods of excessive PM_2.5_ concentration. Another influential variable was the season, as outpatient visits for knee osteoarthritis had a stronger correlation with PM_2.5_ concentration during spring than during summer. This is probably due to the fact that the environmental conditions in spring, when people are more likely to engage in outdoor activities and open windows for ventilation. This may increase the chances of exposure to pollutants. These findings indicate that it is important to be cautious about exposure to air pollutants even in the warm season. Finally, we observed that gender had a strong influence, as the effect of PM_2.5_ was significantly stronger in women than in men. However, the underlying mechanism remains to be explored.

One of the biggest strengths of this study is its large population size and its location in Beijing, which has high air pollution levels and, therefore, is a source of comprehensive and representative data. Additionally, diagnosis was confirmed based on both the ICD-10 code and the corresponding Chinese criteria, so there is a lower risk of bias caused by inaccurate diagnosis and coding inaccuracy.

Some limitations of this study must be mentioned. Firstly, as the city of Beijing did not have a system for comprehensively monitoring all kinds of air pollutants until 2013, for the given study period, data could not be obtained about other pollutants (for example, NO_2_, sulfur dioxide, carbon monoxide, ozone, and particulate matter with an aerodynamic diameter ≤10 μm [PM_10_]). Therefore, we could not determine the independent effect of PM_2.5_. This is an important line of investigation for future studies. Secondly, although several studies have shown that the air pollution data provided by the US Embassy are reliable, the personal exposure data are estimated from a fixed monitor, which is prone to some degree of inaccuracy [46]. Finally, the findings of the subgroup analysis results may have limited statistical power due to the remarkably smaller sample size.

## 5. Conclusions

The present findings show that short-term exposure to PM_2.5_ may be associated with adult outpatient visits for knee osteoarthritis. Importantly, women and elderly people are likely to be more susceptible to the effects of this pollutant. The comprehensive data from this large-scale study make an important contribution to understanding the impact of PM_2.5_ pollution in China. In the future, it would be highly useful to determine the impact of other air pollutants and examine their independent effects.

## Figures and Tables

**Table 1:** Data on outpatient visits for knee osteoarthritis between 1 January 2010, and 31 December 2017, in Beijing, China.

Variable	All Year	Cool Season	Warm Season
Outpatient visits	9,797,446	5,865,689	3,931,757
Sex			
Male(%)	3,565,162 (36.39)	2,140,860 (36.50)	1,424,302 (36.23)
Female(%)	6,232,284 (63.61)	3,724,829 (63.50)	2,507,455 (63.77)
Age(year)			
18–64(%)	6,254,770 (63.84)	3,733,856 (63.66)	2,520,914 (64.12)
≥65(%)	3,542,676 (36.16)	2,131,833 (36.34)	1,410,843 (35.88)

**Table 2:** Daily values of outpatient visits for knee osteoarthritis, PM_2.5_ concentrations, and environmental parameters.

Variable	Mean ± SD	Minimum	Percentile			Maximum	IQR
			25th	50th	75th		
Daily outpatient visits	3354 ± 3700	108	324	2406	4684	30,125	4360
PM_2.5_(μg/m^3^)	86.8 ± 74.3	1.0	33.3	66.5	115.0	537.3	81.7
Daily outpatient visits during the cool season	3457 ± 4074	108	116	2324	4960	30,125	4844
Daily outpatient visits during the warm season	3212 ± 3103	133	942	2492	4287	14,596	3345
Temperature (℃)	14.6 ± 11.3	−14.3	2.6	15.1	24.0	34.5	21.4
Relative humidity (%)	51.8 ± 20.2	8	35	52	68	88	33

IQR: interquartile range, SD: standard deviation.

**Table 3:** Percentage change in the number of outpatient visits for knee osteoarthritis corresponding to a 10-μg/m^3^ increase in PM_2.5_ concentration for various lag structures.

Lag Day	Percentage Change	95% Confidence Interval	*p*
Lag 0 day	1.20	1.20–1.21	<0.001
Lag 1 day	0.61	0.61–0.62	<0.001
Lag 2 day	0.59	0.59–0.59	<0.001
Lag 3 day	0.54	0.54–0.55	<0.001
Lag 0–1 days	1.22	1.21–1.22	<0.001
Lag 0–2 days	1.32	1.31–1.32	<0.001
Lag 0–3 days	1.41	1.40–1.41	<0.001

**Table 4:** Gender-, age-, and season-dependent differences in the percentage change in the number of outpatient visits for knee osteoarthritis corresponding to a 10-μg/m^3^ increase in the same-day PM_2.5_ concentration.

Subgroups	Percentage Change	95% Confidence Interval	^a^ *p*
Sex			
Male	1.36	1.35–1.37	<0.001
Female	1.41	1.40–1.42	<0.001
Age(year)			
18–64	1.28	1.28–1.29	<0.001
≥65	1.59	1.58–1.60	<0.001
Season			
Cool	0.75	0.74–0.76	<0.001
Warm	2.76	2.75–2.78	<0.001

^a^*p* value according to the Z-test for differences between the two risk estimates derived from subgroup analyses.

**Table 5:** Percentage change in the number of outpatient visits for knee osteoarthritis in response to variations in calendar time, temperature, and relative humidity.

Variable	*df*	Percentage Change	95% Confidence Interval	*p* Value
Calendar time	6	1.40	1.39–1.40	<0.001
	7 *	1.41	1.40–1.41	<0.001
	8	1.25	1.24–1.25	<0.001
	9	1.25	1.24–1.26	<0.001
Temperature	5	1.44	1.43–1.45	<0.001
	6 *	1.41	1.40–1.41	<0.001
	7	1.33	1.32–1.33	<0.001
	8	1.37	1.36–1.37	<0.001
Relative humidity	3 *	1.41	1.40–1.41	<0.001
	4	1.42	1.41–1.42	<0.001
	5	1.42	1.41–1.42	<0.001
	6	1.42	1.41–1.43	<0.001

* The degree of freedom (*df*) value used in this study model.

## Data Availability

The datasets generated and/or analyzed during the current study are owned by the government of China and are not publicly available, but are available from the corresponding author on reasonable request.
